# Deaf Child Signers’ Reading Behaviors Are Predicted by Their Bilingual (Signed and Spoken) Vocabularies: Evidence from Eye-Tracking

**DOI:** 10.3390/jemr19040083

**Published:** 2026-08-03

**Authors:** Frances G. Cooley, David Quinto-Pozos

**Affiliations:** 1 Department of Liberal Studies, National Technical Institute for the Deaf (NTID), Rochester Institute of Technology, 1 Lomb Memorial Drive, Rochester, NY 14623, USA; 2 Department of Linguistics, University of Texas at Austin, Patton Hall, 305 E 23rd St Ste 4.304, Austin, TX 78712, USA; davidqp@austin.utexas.edu

**Keywords:** reading, eye-tracking, diverse populations

## Abstract

Deaf children who acquire a signed language at home and at school prior to learning to read are a unique group of developing bilinguals. They are first-language users of a signed language (e.g., American Sign Language; ASL) and second-language users of the written form of the ambient spoken language (e.g., English). Because signed languages do not possess orthographies that are widely used, deaf readers typically read in their second language. This profile stands in contrast to hearing bilingual readers, who read in their first language and, sometimes, their second language. In studies of child readers, vocabulary knowledge is a strong predictor of reading comprehension for both monolinguals and bilinguals. We combine the unique linguistic backgrounds of young signing deaf readers with what is known about vocabulary knowledge and reading, guided by the following research question: Does signed (L1) and spoken (L2) vocabulary knowledge predict reading (eye-gaze) behaviors of deaf children when reading in their L2? We present results from an exploratory pilot eye-tracking study of nine deaf ASL-English bilingual children ages 9–11, examining whether ASL and English vocabulary knowledge predict different aspects of the reading paradigm. Results suggest that ASL vocabulary predicts where-decisions and measures of oculomotor control such as skipping and regressions, while English vocabulary predicts when-decisions and first-pass durations. We suggest these results from this small-scale study provide initial support for the language interdependence hypothesis because deaf child signers’ first-language vocabulary knowledge predicts some of the variance in second-language reading performance.

## 1. Introduction

Reading development in deaf and hard-of-hearing children (hereafter, we use the term ‘deaf’ to refer to individuals who do not access speech sounds without amplification) has often been framed with a primary focus on reduced access to spoken language and its consequences on sound-based phonological processing. Within this framework, successful reading development is assumed to depend heavily on the ability to map written orthography onto spoken language representations, leading many educational and theoretical approaches to prioritize speech-based decoding skills [1]. While this perspective may characterize some deaf readers, particularly those with some access to speech and/or extensive speech training, it does not adequately explain the experiences of deaf children who acquire a natural signed language from an early age and rely on signing over speaking for everyday communication.

Deaf child readers who acquire a full, natural signed language prior to learning to read represent a unique group of bilinguals. These children typically develop linguistic competence in a visual–manual language (e.g., American Sign Language in the U.S.; ASL) and learn to read the written form of the ambient spoken language as a second language (e.g., English in the U.S.). Unlike hearing bilinguals, who often read in both their first (L1) and second language (L2), deaf child signers read exclusively in their L2 because signed languages have no conventional, community-used orthographies. This raises a fundamental question regarding reading development in deaf populations: How does language knowledge in a visual–manual L1 support the development of reading in a written L2? More specifically, what types of relationships exist between a deaf signer’s L1 and their written/spoken L2?

One promising approach to understanding this question leverages data from eye-tracking methodologies, which provide a powerful window into the moment-to-moment decisions that occur during reading. Readers do not move their eyes in a smooth, straight line across text. Instead, the eyes perform a series of saccades (e.g., high velocity movements between two points) and fixations (e.g., moments where the eyes are still [2]). Eye-movements can be further categorized into two partially dissociable processes: when- and where-decisions [3]. When-decisions reflect how long a reader’s eyes should remain in place before moving and are commonly indexed via timing-based measures, such as fixation durations. These measures are most commonly reflective of lexical retrieval, with shorter fixation durations reflecting faster, more efficient extraction of meaning from text [4]. In contrast, where-decisions reflect how readers guide their eyes through text, including which words are skipped, reread, or selected for an upcoming fixation [3,5,6,7]. Together, these measures provide insight into both lexical processing, visual-attentional processing, and oculomotor control, offering a window into the efficiency of the reading system and the information that readers attend to in support of successful comprehension (see [2] for a review).

A growing body of work has shown that skilled deaf adult readers, particularly those who are early signers, exhibit a highly efficient reading profile. Compared to hearing readers, they have been shown to demonstrate faster reading, increased word skipping, and reduced reliance on regressions and rereading without a negative impact on comprehension [8,9,10,11,12]. In addition, multiple studies report that deaf readers possess an expanded visual perceptual span during reading, with evidence for enhanced parafoveal processing both to the right [6,13,14] and to the left of fixation [15]. At the same time, skilled deaf child and adult readers who are early signers do not appear to rely on speech-based representations to the same extent as hearing readers [8,16,17,18]. These findings suggest that deaf readers, particularly those with access to a signed language, depend on factors other than sound-based coding to comprehend written language.

Developmental evidence suggests that deaf signers’ reading efficiency may emerge early in reading acquisition. Studies of deaf children who had early access to a signed language indicate that more skilled deaf child readers are capable of extracting orthographic and semantic information from parafoveal vision, with limited evidence of sound-based phonological involvement [19]. Skilled deaf child readers also have been shown to exhibit an enhanced rightward perceptual span [20] and they appear to rely less on speech-based codes and regressing back in text when resolving errors in text compared to hearing readers [16]. Some sound-based phonological sensitivity may also be gained through the process of reading for particularly skilled deaf readers [8,21]. Together, these findings suggest that deaf child signers may be developing the foundations of an efficient reading system from an early age despite not primarily engaging in sound-based phonological decoding. However, while these studies reflect a transition to skilled, efficient reading in deaf children, they do not yet explain the linguistic mechanisms that support efficient and successful reading comprehension.

### 1.1. Bilingualism and Vocabulary Knowledge

One promising approach to answering the question of reading development in deaf children comes from the research on hearing bilingual populations. According to the language interdependence hypothesis [22,23], conceptual knowledge and linguistic skills can transfer across languages, even when those languages differ in modality. Applied to deaf child signers, this framework predicts that a stronger L1 foundation in a signed language may support acquisition of and reading performance in a written L2. Several important components of sign language knowledge may support L2 reading in deaf child signers, including phonological awareness [24], orthographic sensitivity and fingerspelling knowledge [25,26,27], and vocabulary knowledge [28,29,30,31,32,33,34,35,36,37,38].

Vocabulary knowledge and understanding of what words mean in a language is imperative for successful reading, and research with hearing bilinguals consistently demonstrates that robust L1 lexical knowledge supports L2 (reading) development [39,40,41,42,43,44]. Readers with stronger L1 vocabularies and richer word representations in their mental lexicons tend to exhibit more efficient lexical processing in their L2, facilitating faster word recognition and more efficient integration of information across sentences [39,41,42]. These advantages are thought to arise because well-developed L1 lexical representations provide a foundation for cross-language activation and transfer, allowing bilingual readers to leverage existing semantic and conceptual knowledge when processing their L2 [40,43,44]. Studies further suggest that high-quality L1 lexical representations support syntactic and semantic integration during L2 processing, resulting in better comprehension outcomes and more efficient allocation of cognitive resources [42]. While prior work has explored the relationship between vocabulary knowledge and reading achievement in deaf populations [28,29,30,31,32,33,34,35,36,37,38], there have been no empirical investigations into how L1 and L2 vocabulary knowledge shape the real-time processes involved in reading for young deaf bimodal bilinguals.

### 1.2. The Present Study

This study was developed as a pilot to explore the relationship between L1 ASL knowledge and L2 English knowledge, while also collecting data on other linguistic and cognitive tasks, and online measures of reading in skilled deaf child readers. Considering that lexical information is primarily extracted during fixations [4], we hypothesized that English vocabulary knowledge would be most strongly predictive of when-decisions (first-pass time, total time) and that readers with stronger English vocabulary would have shorter fixation times. In addition, considering visual information primarily guides where-decisions and eye-movement control [45] and the visual linguistic processing expertise of skilled deaf adult readers who learned ASL before learning to read [9], we hypothesized that stronger ASL knowledge would predict more efficient use of visual information. In the context of child readers who are signers, these hypotheses would suggest that more skilled reading will be associated with fewer skips along with fewer regressions, reflecting more precise use of eye-movements during the first pass, reducing the need to look back in text.

## 2. Materials and Methods

### 2.1. Participants

Data from nine deaf children (mean age 9 years, 11 months, range 9.5–11.7; five female) are included in this analysis. Two additional deaf children participated in portions of the data collection, but no eye-tracking data were collected from them due to scheduling challenges. Of the nine children, parents reported that seven were profoundly deaf (70–90 db loss), one had a moderate-to severe hearing loss (55–70 db), and one had a moderate hearing loss (40–55 db loss). All children were reported to have signing relatives at home, and eight of the nine had at least one deaf parent who is a signer of ASL. The cause of deafness for eight of the nine children was reported as “genetic/hereditary”. None of the children were cochlear implant users, and two were reported to use a hearing aid, although no information was provided about how often such a device was used. Four parents reported that their children had participated in speech and language therapy. Seven parents reported that their child engaged in literacy practices before entering school. See Table 1 for socio-economic data (we do not include SES information in our models due to the small sample size.).

All children attended a bilingual-bicultural (ASL–English) school for the deaf where teachers, educational staff, and students use ASL, and all the data collection was conducted at the school. Five of the children did not complete all blocks (*n* = 3) of the homophone foil reading task. Three children completed two blocks, and two completed one block each. Thus, four children completed all blocks (total of 36 sentences). Statistical models were chosen, in part, to manage different amounts of eye-tracking data across child participants.

### 2.2. Behavioral Measures

Children were administered measures of reading comprehension (*PIAT-R*), word reading fluency (*TOSWRF*), non-verbal fluid reasoning and problem solving (*KBIT-2 matrices*), working memory (*OOO*), fingerspelling (VL2 Fingerspelling Test), and vocabulary (*ASL-VT*, *E-VT*). Such measures were randomized throughout multiple sessions (on different days) per participant. Additionally, during one or more sessions children’s eye-gaze behaviors were tracked while they read isolated sentences (see below for details).

*Peabody Individual Achievement Test-Revised (PIAT-R):* Reading level was assessed via the reading comprehension subtest of the PIAT-R [46] (test–retest reliability r = 0.88). For this task, participants are asked to read individual sentences then match the sentence with a picture from an array of four. Items increase in difficulty by incorporating complex vocabulary items and syntactic structure. Participants completed the task when they answered five of seven consecutive items incorrectly. Standard scores are reported.

*Test of Silent Word Reading Fluency (TOSWRF):* Word reading fluency was measured by the TOSWRF [47] (test–retest reliability r = 0.84–0.92). In this task, participants were presented with strings of concatenated words without spaces and were instructed to identify word boundaries by marking separations between words within a timed interval.

*Kaufman Brief Intelligence Test (KBIT)-2:* Nonverbal skills were assessed via the KBIT-2 [48] (test–retest reliability r = 0.85). Participants were presented with an array of visual objects and were instructed to select which target from a group of distractors best fit with the array. This task concludes when participants have four consecutive errors or complete the task.

*Odd One Out (OOO):* The Odd One Out task [49] measures executive-loaded visuo-spatial working memory (test–retest reliability r = 0.81 [50]). Participants are presented with three shapes; two are identical, one does not match the others. After exactly 2 s, three different shapes replace the target array. Participants are asked to identify the position of the odd-one-out shape from the target array.

*VL2 Fingerspelling Test*: Participants completed the VL2 Fingerspelling test [51] as a metric of their sensitivity to sign-based spelling. On each trial, participants viewed a video of a native signer producing a fingerspelled real English word or phonotactically legal pseudoword. Participants were asked to reproduce the fingerspelled item in writing and indicate whether it was a real or fake word in English. Participants were administered 70 items and were scored as percentage correctly reproduced.

*American Sign Language Vocabulary Test (ASL-VT) 1–4*: ASL vocabulary knowledge was measured by the ASL-VT, subtests 1–4 [52]. Each subtest is designed to test a different aspect of vocabulary knowledge and reflects different strengths of form-to-meaning mapping. There are two sets of 40 age-appropriate vocabulary items each, A and B, which are used alternatively to aid with counterbalancing and reduce learning effects when the test is administered regularly. To further reduce learning effects across each subtest during the test administration—all subtests use the same target signs—the ASL-VT is designed so that participants complete the first three subtests in a specific order with item set A and the fourth subtest with item set B (or vice versa). All sign stimuli were produced by native deaf signers. Subtests are administered in the following order:

1. Form recall (strong mapping)—Participants are asked to produce an ASL sign in response to a picture prompt.
2. Form recognition (weak mapping)—Participants are asked to match a picture prompt with one of four ASL signs.
3. Meaning recall (strongest mapping)—Participants are asked to produce three ASL signs in response to a sign prompt.
4. Meaning recognition (weakest mapping)—Participants are asked to match an ASL prompt with one of four pictures.

*English Vocabulary Test (E-VT) 1–4*: English vocabulary knowledge was measured by the E-VT [33,52], subtests 1–4. The structure of the E-VT matches that of the ASL-VT. Each subtest is designed to test a different aspect of vocabulary knowledge and reflects different strengths of form-to-meaning mapping. Like the ASL-VT, the entire test is designed so that participants complete the first three subtests with one list of 45 vocabulary items; participants see a new list of 45 items on the fourth subtest. This structure is designed to aid with counterbalancing and reduce learning effects across each subtest:

1. Form recall (strong mapping)—Participants are asked to produce a written English word in response to a picture prompt.
2. Form recognition (weak mapping)—Participants are asked to match a picture prompt with one of four written English words.
3. Meaning recall (strongest mapping—Participants are asked to produce three written English words in response to a written English prompt.
4. Meaning recognition (weakest mapping)—Participants are asked to match a written English prompt with one of four pictures.

**Figure 1:**
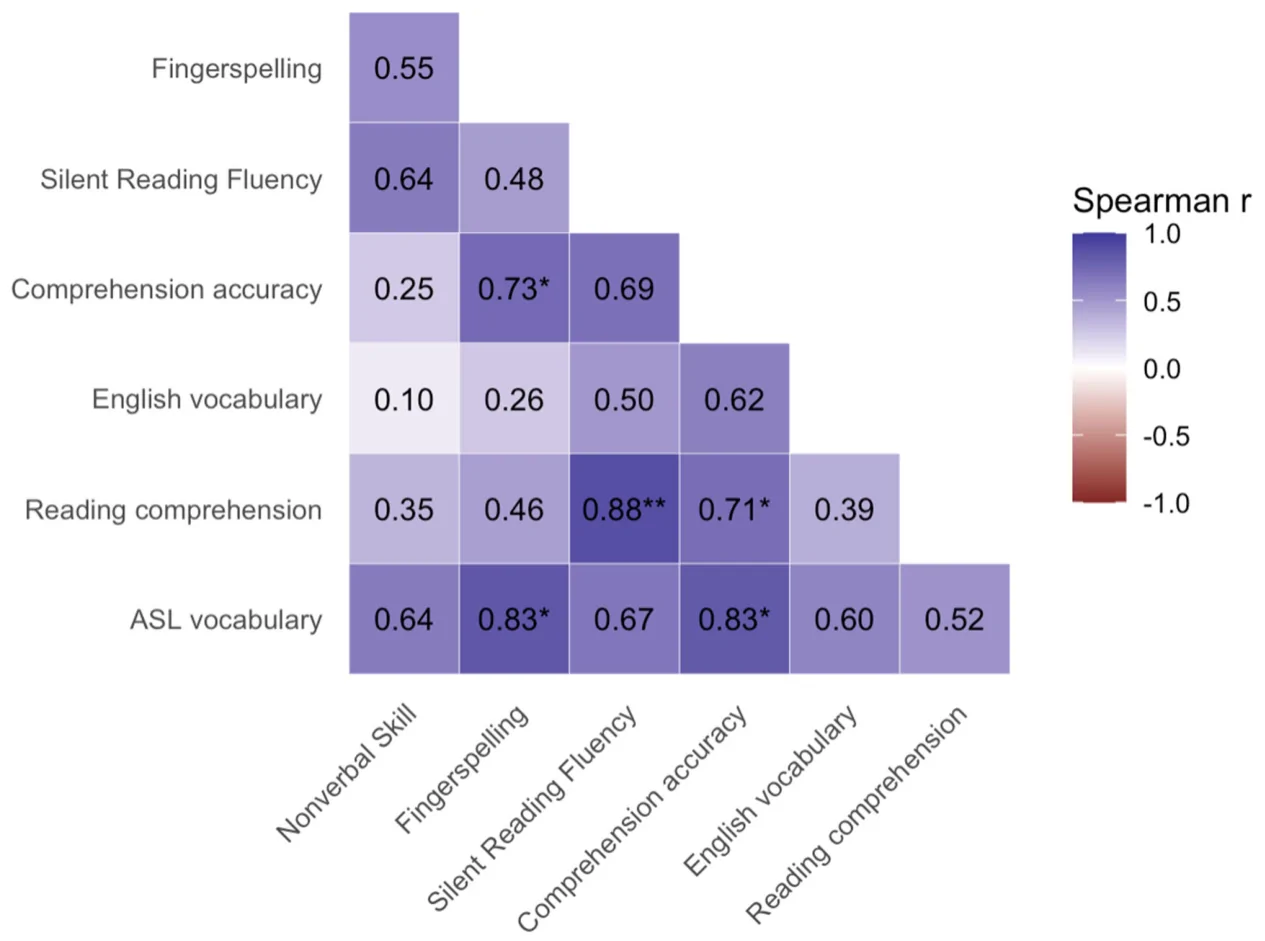
Correlational matrix between all behavioral measures. Note: * = *p* < 0.05; ** = *p* < 0.01; All behavioral measure scores are scaled and centered; Comprehension Accuracy = proportion of correct responses to comprehension questions on the eye-tracking task; ASL Vocabulary = averaged scores on the four subtests of the ASL-VT; English Vocabulary = averaged scores on the four subtests of the E-VT; Reading Comprehension = raw score on the independent measure of reading comprehension (PIAT); Silent Reading Fluency = score on TOSWRF; Fingerspelling = score on VL2-Fingerspelling test; NonVerbal Skill = score on KBIT.

See Table 2 for behavioral measurement information, Table 3 for a breakdown of ASL- and E-VT scores, and Figure 1 for a correlational matrix of all behavioral measures.

### 2.3. Experimental Eye-Tracking Task

Participants completed the homophone foil reading task [16,53] while their eye movements were recorded with an EyeLink 1000 Portable DUO (SR Research, Ottowa, Canada) sampling at 1000 Hz. Sentences were viewed binocularly; all analyses reported here use the right-eye record. Before calibration, participants were instructed to read naturally for meaning while resting their head on a chin and forehead support positioned 60 cm from the monitor. Stimuli were displayed in 12-point Courier New using the University of Massachusetts Amherst Eye-Tracking Lab software EyeTrack (v7.10k). A horizontal three-point calibration was performed at the outset of the session and was checked frequently throughout. Validation values were considered satisfactory if values were 0.30 or less. Calibration was repeated whenever accuracy drifted, when the participant noticeably shifted position, or when drift correct was visibly shifted.

Each trial began when the participant fixated a gaze-contingent trigger. Once fixation was registered, the sentence appeared, with the first character aligned to the trigger location so that readers began at the start of the sentence. Target words appeared in one of three conditions (correct, homophonic error, nonhomophonic error; Experiment 3 in [53]). Correct targets and their corresponding foils were balanced for frequency, crossing high- versus low-frequency targets with high- versus low-frequency foils so that items were evenly distributed across the four frequency pairings. Participants read the same sentence frames across conditions, with target-word condition varying by list. Each participant read up to 108 experimental sentences presented in three blocks of 36, interspersed with filler sentences. To reduce order effects, participants were randomly assigned to a starting block and the remaining blocks followed in a counterbalanced sequence. Due to scheduling constraints, only four participants completed all three blocks; three participants completed two blocks, and two participants completed only one. To encourage reading for comprehension, yes/no questions about the previous sentence were presented approximately every fourth trial, following both experimental and filler items. Proportion of correct responses to comprehension questions on the experimental eye-tracking task, which were interspersed with sentence reading to encourage attention to the content of sentences, is included in the correlational analysis as Comprehension Accuracy (see Table 1 for mean [sd] and range). See [16] for additional detail on how a different group of deaf child signers performed on the experimental aspect of this paradigm.

For the analyses reported here, we restricted the dataset to the correct-condition sentences and extracted word-level measures for all words within each sentence, excluding the sentence-initial and sentence-final words. This allowed for an analysis of reading behaviors without an error in the sentence, while increasing the number of word observations. Data cleaning was performed by software from the UMass Amherst eye-tracking lab. Raw .asc datafiles were cleaned and compiled into .da1 files using Robodoc, which automatically excludes blinks. Finally, specific eye-movement measures were calculated and compiled by EyeDry. We removed all first-pass fixations greater than 1000 ms and less than 60 ms, resulting in the loss of 10 observations (~1%). The final analysis included 909 observations from nine child readers on 151 unique words (frequency: mean 11.2, SD 2.5 [log HAL frequency [54]]; length = mean 4.7, SD 1.8).

To assess online reading behaviors, we tested four common eye-movement metrics: Skipping probability (binary; whether the word was skipped or fixated during the first-pass), regression probability (binary; whether the word was regressed back to in a second pass), first-pass time (continuous; how long the word was fixated during the first pass; ms), and total time (continuous; how long the word was fixated cumulatively during all passes; ms). See Table 4 for descriptive statistics of reading behaviors. Due to the limited dataset, we did not perform any automatic exclusions of participants.

### 2.4. Analyses

For each measurement, we conducted mixed-effect (generalized) linear regression models for word skipping, first-pass fixation time, regressions in, and total reading time including random effects of participant and word item, testing the role of the (centered and scaled) composite scores on the ASL-VT and E-VT. Word length, word frequency, and participant age are also included in the models as control.

Full models including ASL-VT and E-VT composite scores, word length, word frequency, and participant age as fixed effects. Participant and word token as random effects were run and evaluated using lme4 [55], and lmerTest [56] and effect sizes were calculated using effectsize [57] in R version 4.3.3 (29 February 2024).

### 2.5. Model Testing

For the binomial models (skipping and regressions), simulation-based randomized quantile residuals were generated using the DHARMa package [58] with 1000 simulations. Overall model fit was evaluated using tests of residual uniformity, dispersion, and excess outliers. Dispersion was assessed using simulations conditional on the random effects. Because the outcomes were trial-level Bernoulli variables, residuals were also aggregated separately by participant and word token to evaluate whether model misspecification was structured by either grouping factor. Shapiro–Wilk tests of standardized residuals and Breusch–Pagan tests of nonconstant error variance were also conducted.

The binomial models showed no evidence of misspecification. Tests of conditional dispersion, residual uniformity, excess outliers were nonsignificant, and both models converged. The skipping model produced a nonsingular fit, but the initial regression model produced singular fit. Removing the random effect of word token resolved singularity, and the final model converged with a nonsingular fit.

Models fitted to first-pass and total-reading times showed significant residual nonnormality and heteroscedasticity (*p* > 0.05). Fixation times were therefore log-transformed and tested, which resolved the heteroscedasticity for both measures without changing the pattern of effects. Due to the similarity in overall effects with and without transformation and the difficulty interpreting coefficient values in log-transformed models, we maintain raw fixation duration data for analyses reported below, which is in line with what has been reported in the literature (see [59] for a review). See Table 5 for model output results. Trial-level data, stimuli, and the code used for analyses can be found here: https://osf.io/g85sh.

## 3. Results

### 3.1. ASL Vocabulary Knowledge

**Figure 2:**
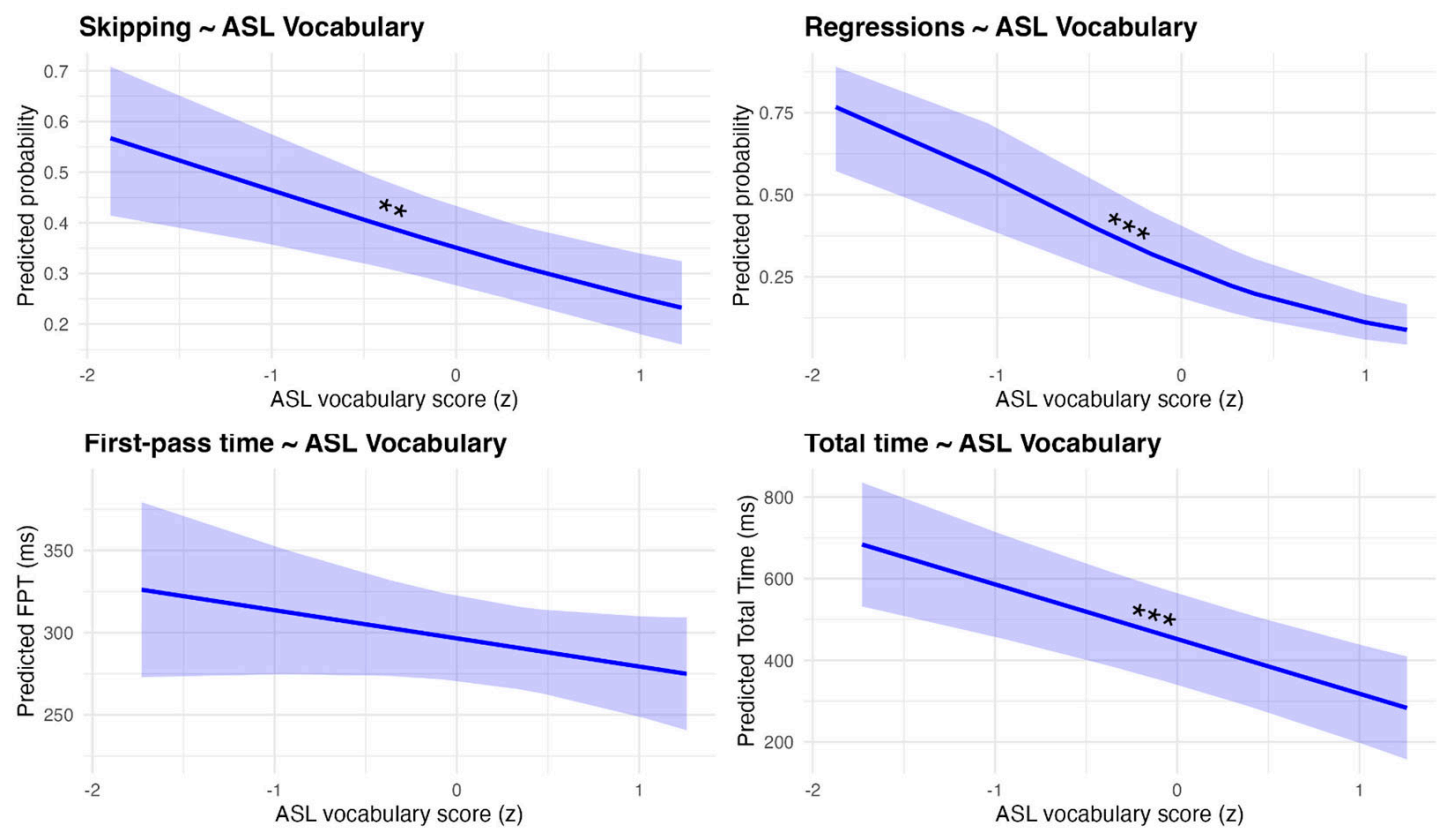
Eye-tracking measures by ASL Vocabulary Knowledge. Note: ** = *p* < 0.01, *** = *p* < 0.001; Lines reflect lines of best fit for residuals from mixed-effects models; shaded region represents 95% confident interval.

ASL vocabulary knowledge significantly predicted deaf child readers’ skipping (z = −3.17, *p* < 0.01; d = −0.44, 95% CI = [−0.72–−0.17]), regressions into words (z = −5.09, *p* < 0.001; d = −1.02, 95% CI = [−1.41–−0.63], and total time (z = −4.88, *p* < 0.001, d = −0.32 [95% CI = [−0.45–0.19]. See Figure 2.

### 3.2. English Vocabulary Knowledge

**Figure 3:**
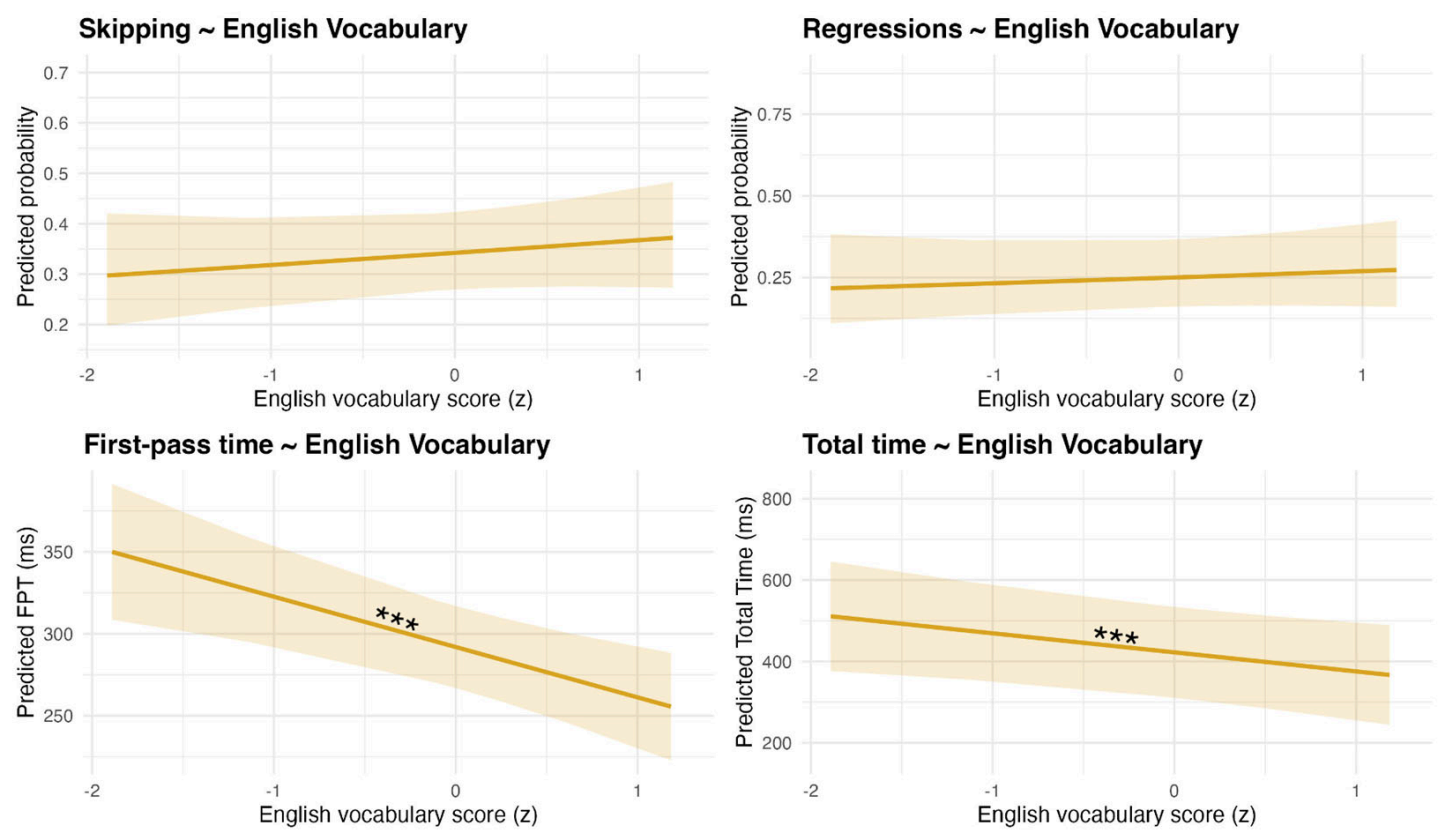
Eye-tracking measures by English Vocabulary Knowledge. Note: *** = *p* < 0.001. Lines reflect lines of best fit for residuals from mixed-effects models; shaded region represents 95% confident interval.

In contrast to ASL, English vocabulary knowledge predicted deaf child readers’ first-pass (t = −3.43, *p* < 0.001, d = −0.2, 95% CI = [−0.32–−0.09]) and total reading times (t = −2.27, *p* < 0.001; d = −0.12, 95% CI = [−0.23–−0.02]). See Figure 3.

## 4. Discussion

This exploratory pilot study aimed to examine whether L1 ASL vocabulary knowledge and L2 vocabulary knowledge relate to eye-movement behaviors during online reading for child deaf readers. We report analyses of vocabulary knowledge and eye-movement measurements from nine deaf children who use a signed language at home and attended a bimodal bilingual school for the deaf while reading words in age-appropriate sentences. Previous eye-tracking studies with deaf children who are native or early signers suggest that they engage in qualitatively different eye-movement strategies when reading [13,16,19,20]. Though the relationship between L1 and L2 vocabulary knowledge on reading skill has been theorized and correlations between the two have been reported for deaf child signing readers [28,29,30,31,32,33,34,35,36,37,38], no studies to date test the relationship between ASL and English vocabulary knowledge and online reading behaviors in deaf bimodal bilingual children. Below, we summarize the findings presented here and speculate on what these data suggest if the patterns were to hold up with a larger dataset with more statistical power.

The results presented here reflect a notable dissociation between the role of signed and spoken language vocabulary knowledge during reading for deaf child signers. ASL vocabulary knowledge related to where-decisions during reading such that deaf children with overall stronger ASL vocabulary knowledge progressed more carefully through text: They skipped fewer words and depended less on regressing, which ultimately correlated with better performance on the comprehension questions (skip: *p* < 0.05; regressions: *p* < 0.001). In contrast, English vocabulary knowledge predicted timing-based decisions first-pass and total fixation durations. Together, these findings suggest that vocabulary knowledge in both of a deaf child’s (signed and spoken) languages support reading behaviors, and each language may support different components of the reading process.

Importantly, these findings extend beyond a simple relationship between vocabulary knowledge and reading skill. Deaf signers are unique bilinguals: They read exclusively in an L2 that differs in structure and modality from their L1. Unlike hearing bilinguals who (typically) engage in two spoken languages, deaf signers share few modality-related structures across their two languages. Nevertheless, ASL vocabulary knowledge predicted some of the variance in online English reading behavior and correlated with comprehension questions on the experimental reading task (Comprehension Accuracy; r = 0.83; *p* < 0.05), reading levels (PIAT; r = 0.52), and English vocabulary knowledge (EVT; r = 0.6). ASL vocabulary knowledge also predicted total reading time (*p* < 0.001) and marginally predicted first-pass reading time (*p* = 0.065), a finding that may become significant with sufficient statistical power. These findings may provide preliminary support for the application of the language interdependence hypothesis [22,23] to bimodal bilingualism in deaf signing children. Results suggest that transfer between languages does not require a shared phonological or orthographic code, but instead that stronger signed language knowledge may support broader linguistic processes involved in successful reading.

The relationship between ASL vocabulary knowledge and where-decisions is particularly noteworthy given previous findings that skilled deaf adult readers often exhibit highly efficient eye-movement strategies characterized by increased skipping and reduced rereading [8,9,10,11,12,13]. At first glance, the present finding that stronger ASL vocabulary knowledge predicted less word skipping may appear inconsistent with the adult literature. However, this pattern may reflect an earlier developmental stage in the emergence of skilled reading behaviors. One possibility is that high skipping rates in developing and skilled readers reflect different underlying processes. In skilled deaf adult readers, word skipping is a highly efficient behavior: Skipping is strongly linked to the visual and linguistic content of text [9,10], and deaf adults show more resilient saccade targeting following skips compared to hearing adult readers [60] without a negative impact on overall comprehension. In contrast, for developing deaf readers, skipping appears less consistently guided by lexical and parafoveal information and may instead reflect an earlier stage of learning how to best leverage text information [9,10] and develop efficient saccade targeting [55]. Indeed, the results from this pilot study suggest that reading behaviors are not strongly tied to word-level characteristics (e.g., word length and frequency) for young deaf readers, but instead appear linked to other linguistic and visual factors. From this perspective, the negative relationship between ASL vocabulary knowledge and skipping may reflect an earlier stage of more selective (i.e., careful) and controlled first-pass reading. Importantly, skipping and regressions decreased as ASL vocabulary knowledge increased. Higher rates of skipping and regressions also negatively correlated with online comprehension accuracy (*p* < 0.05 and *p* < 0.001, respectively). Together, these findings support the hypothesis that children with stronger ASL vocabulary knowledge may make more selective skipping decisions during first-pass reading and rely less on regressions to repair comprehension difficulties or engage in confirmatory comprehension monitoring. This more careful and controlled reading pattern may, in turn, support better comprehension. This pattern warrants further investigation in a larger dataset and a wider range of participant ages.

**Figure 4:**
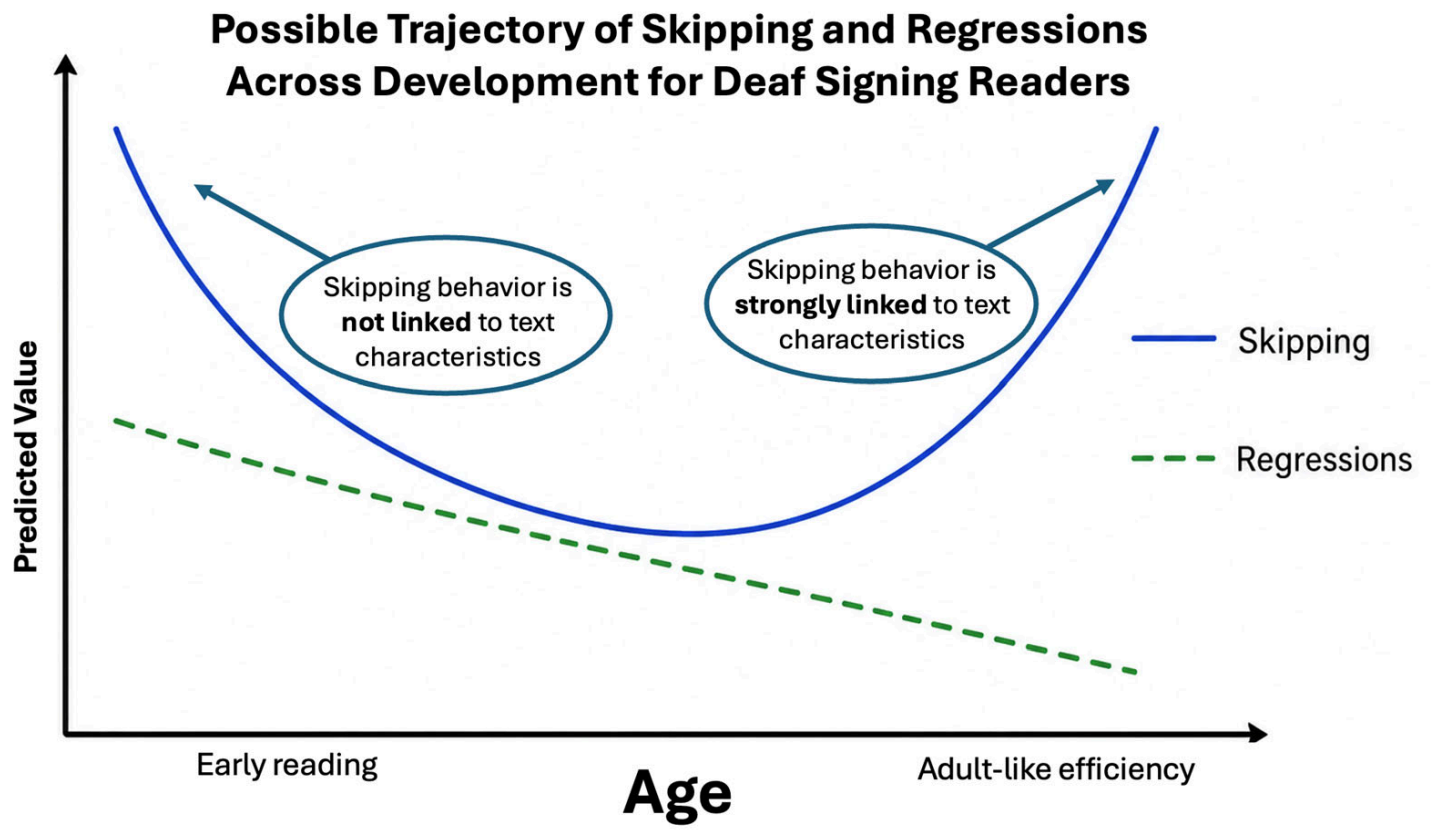
Possible trajectory of skipping and regressions for deaf signing readers.

Our hypothesis is that deaf readers may demonstrate a U-shaped curve of skipping behaviors in conjunction with a relatively stable decrease in regressions throughout the development of reading skills. These pilot results suggest that, early in reading development, deaf readers begin to read with high rates of skipping and regressions. As signed (and spoken language) knowledge is gained, readers likely develop more precise reading strategies whereby they skip less (e.g., progress more carefully during the first-pass) in conjunction with fewer regressions (e.g., less reliance on second-pass reading for repair processes), reflecting the development of more efficient eye-movement strategies as readers progress through text. Once deaf readers have transitioned from learning to read to reading to learn and achieve adult-like reading efficiency, their skipping behaviors become more directly linked to the content of words (and influenced by frequency and length characteristics more) and increase without needing to regress [9,10]. By using the content of the text more efficiently, skilled deaf readers may ultimately achieve superior integration of language and textual information. Future analyses of older adolescent deaf readers and/or a longitudinal study would be necessary to evaluate the validity of this proposal. See Figure 4 for a visual representation of this relationship.

The present findings also connect to prior work showing that deaf child and adult signers demonstrate enhanced visual attention and parafoveal processing during reading [13,14,15,19,20,21,56]. Previous studies suggest that deaf signers extract more useful information from the parafovea and exhibit expanded perceptual spans compared to hearing readers. The current results raise the possibility that stronger ASL vocabulary knowledge may support these visual–attentional mechanisms during reading development. Specifically, stronger visual–gestural language experience may facilitate more efficient allocation of visual attention across text. As reading skill is developed, deaf signers may become increasingly able to take advantage of their wider spans [6,12,13,14,15,19,20] and increased attention to the periphery [61]. This interpretation aligns with broader developmental research suggesting that skilled reading involves increasingly efficient coordination between parafoveal processing, lexical access, and oculomotor control over time [62,63,64,65]. While results from the present study cannot be used directly to assess the role of the perceptual span and parafoveal processing on deaf children’s reading behaviors, the findings do suggest that signed language knowledge may influence online reading through pathways related to visual attention and oculomotor control.

In contrast, English vocabulary knowledge predicted timing-based when-decisions (note, however, the small effect sizes for these measures). This finding aligns with previous work and theories regarding lexical access during reading: Because very little visual (and, therefore, linguistic) information can be processed during saccades [2,66], lexical information is extracted during fixations, with shorter fixation durations reflecting faster lexical retrieval and tighter connection between visual form and meaning [4]. Therefore, this result supports claims that stronger L2 vocabulary knowledge is connected to the extraction of L2 semantic information from written English.

At the same time, ASL vocabulary knowledge was (at least, numerically) related to all eye-movement measures reported here, and L1 ASL and L2 English vocabulary knowledge are correlated for this group (r = 0.58). This finding further reflects deaf child signers’ bilingualism: more robust semantic knowledge in an L1 may help the acquisition of semantic knowledge in an L2 and overall language ability may transfer from first- to second languages. These findings seem to provide support for the language interdependence hypothesis, which suggests that stronger knowledge in an L1 relates to L2 performance [22,23]. Stronger semantic and conceptual knowledge in an L1 may help support the acquisition and organization of semantic knowledge in an L2, even when the two languages differ in modality. Importantly, however, the present findings suggest that transfer may not operate uniformly across all aspects of reading. Instead, L1 and L2 vocabulary knowledge may support different components of the reading system in partially dissociable ways, with signed language knowledge relating more strongly to visual guidance and oculomotor control and spoken-language vocabulary relating more strongly to lexical retrieval during fixations. These findings also align with previous hypotheses of shared cognitive resources being allocated across a deaf bilingual’s two languages [28,29,30,31,32,33,34,35,36,37,38].

Finally, though this study primarily focused on vocabulary knowledge, it is important to note that vocabulary knowledge is by no means the one and only predictor of reading behaviors, especially in deaf child signers. Due to the exploratory nature of this study and a small sample size, we only performed statistical analyses with vocabulary data; however, one particularly important behavioral measure warrants additional comment based on the correlational analysis of all behavioral measures: Fingerspelling (see Table 4). Scores on VL2 Fingerspelling task were significantly correlated with ASL-VT composite scores (r = 0.83, *p* < 0.05) and comprehension accuracy on the experimental reading task (r = 0.73, *p* < 0.05). Fingerspelling has been repeatedly theorized to serve as a bridge between signed language and reading [25,26,27], and the strong correlational evidence that fingerspelling contributes to text reading provides support for such claims. These results further emphasize that fingerspelling remains an important avenue for future investigations of the development of reading skill in deaf children.

Though the results presented here reflect promising early patterns, several limitations should be acknowledged. First, these results are from a very small sample size and effect sizes remain modest (if not weak) and thus should therefore be interpreted as preliminary and exploratory. Second, the current analyses do not permit causal conclusions regarding the role of ASL or English vocabulary knowledge in reading development, particularly considering that all analyses were performed on the same group of readers. Instead, ASL vocabulary knowledge may index broader early language access, bilingual language experience, and protection from possible effects of language deprivation (for those children with delayed exposure to a signed language) rather than vocabulary knowledge alone. Future work with larger samples is needed to disentangle the role of vocabulary breadth (e.g., overall size of the mental lexicon in each language), vocabulary depth (e.g., degree to which lexical entries are connected and co-activate one another in each language), language exposure, and broader bilingual experiences and reading practices in shaping online reading behaviors. Finally, while we make the ASL L1/English L2 distinction repeatedly, we acknowledge that the line between L1 and L2 can be blurry, especially for deaf children. Instead, it may be more appropriate to think of ASL as the primary in-person language experienced by deaf individuals while English serves as the primary text-based language. This relationship between languages may also have an impact on language dominance for this group, which warrants additional future investigation as well.

Future investigations would also benefit from a deeper examination of the mechanisms through which signed and spoken-language knowledge support reading development. In particular, evaluations of semantic network structure, visual attention allocation, and lexical connectivity across languages may prove especially informative. Additional predictors not examined within the statistical analyses, including fingerspelling ability, print spelling ability, working memory, and nonverbal cognitive skills, may contribute meaningfully to successful reading development in deaf bilingual readers. Finally, cross-linguistic evaluations of deaf readers of other scripts (e.g., transparent orthographies, logographic scripts, syllabaries) are necessary to evaluate how the linguistic and visual properties of text impact these behaviors in deaf readers.

## 5. Conclusions

The present findings from this exploratory pilot study suggest that efficient reading development in deaf child signers may emerge through bilingual visual-language pathways that differ fundamentally from frameworks grounded in evidence from hearing mono- and bilingual readers. Despite reading exclusively in their L2, deaf child signers demonstrated relationships between L1 signed and L2 spoken vocabulary knowledge and online English reading behaviors. Though the generalizability of these findings is limited by the sample size, these results further support the view that successful print reading can emerge through multiple linguistic pathways and that strong early access to a natural signed language may play an important role in supporting successful reading development in deaf children.

## Figures and Tables

**Table 1:** Demographic, socio-economic, and general language practice information.

SES Factor	Category	Value
Maternal education	Associate degree	2 (25.0%)
	Bachelor’s degree	2 (25.0%)
	Graduate degree	4 (50.0%)
Paternal education	Less than high school	2 (25.0%)
	Some college or associate degree	2 (25.0%)
	Graduate degree	4 (50.0%)
Annual household income	Less than $50,000	2 (25.0%)
	$50,000–$99,999	3 (37.5%)
	$100,000 or more	3 (37.5%)

Note: Only eight parent surveys were collected.

**Table 2:** Mean performance on behavioral measures.

Measure	PIAT	TOSWRF	KBIT	OOO	Fingerspelling	Comprehension Accuracy
Mean (SD)	85.6 (17.5)	95.1 (6.6)	117.3 (13.7)	13.56 (3.2)	86.79 (5.5)	75.82 (12.6)
Range	55–117	77–104	94–133	9–18	0.78–0.95	59–97

Note: PIAT, TOSWRF, KBIT—Standard Score (SD); OOO—raw score (SD); Fingerspelling—% correct (SD); Comprehension Accuracy = mean (SD) response during experimental eye-tracking task.

**Table 3:** Percent correct on the ASL- and English Vocabulary Tests.

Measure	VT1	VT2	VT3	VT4	Composite
ASL	82.92 (8.6)	89.17 (7.7)	73.87 (13)	95.36 (0.9)	85.39 (0.06)
English	68.02 (13.2)	82.22 (8.5)	64.61 (18.3)	85.43 (8.8)	75.07 (0.1)

Note: Mean (SD); Composite score from all subsets used in all analyses reported below.

**Table 4:** Descriptive statistics for eye-movement measures.

Measure	Skipping	First-Pass Time (ms)	Regression Probability	Total Time (ms)
**Mean (SD)**	0.39 (0.49)	315.76 (187.82)	0.28 (0.45)	438.99 (369.2)

**Table 5:** Regression model output: measure ~ ASL vocabulary + English vocabulary. Bold items reflect significant effects.

	Skipping				Regressions in				First-Pass Time				Total Time			
*Predictors*	*Est.*	*SE*	*z*	*p*	*Est.*	*SE*	*z*	*p*	*Est.*	*SE*	*z*	*p*	*Est.*	*SE*	*z*	*p*
(Intercept)	0.53	0.10	−3.52	**<0.001**	0.37	0.10	−3.53	**<0.001**	291.49	12.96	22.49	**<0.001**	419.58	57.07	7.35	**<0.001**
ASL VT	0.62	0.09	−3.17	**0.002**	0.32	0.07	−5.09	**<0.001**	−16.53	11.57	−1.43	0.154	−129.40	26.51	−4.88	**<0.001**
E VT	1.12	0.14	0.90	0.368	1.10	0.18	0.61	0.545	−30.70	8.94	−3.43	**0.001**	−46.87	20.66	−2.27	**0.024**
Length	1.00	0.16	−0.02	0.988	0.93	0.14	−0.48	0.629	−5.36	9.56	−0.56	0.575	−24.25	19.26	−1.26	0.208
Frequency	0.88	0.15	−0.77	0.440	0.78	0.11	−1.70	0.088	−4.87	10.37	−0.47	0.639	−18.37	19.97	−0.92	0.358
Age	1.60	0.18	4.21	**<0.001**	0.58	0.09	−3.36	**0.001**	−30.87	8.52	−3.62	**<0.001**	−177.85	21.06	−8.45	**<0.001**
**Random Effects**																
σ^2^	3.29				3.29				17,884.07				104,607.37			
τ_00_	1.12 _token_				0.54 _subj_				1668.77 _token_				1459.10 _token_			
	0.11 _subj_								863.68 _subj_				24,303.03 _subj_			
ICC	0.27				0.14				0.12				0.20			
N	8 _subj_				8 _subj_				8 _subj_				8 _subj_			
	175 _token_								155 _token_				161 _token_			
Observations	889				568				539				630			
Marginal R^2^/Conditional R^2^	0.102/0.347				0.198/0.311				0.064/0.180				0.202/0.359			

## Data Availability

Data and analysis scripts are available via OSF (https://osf.io/g85sh).

## Abbreviations

The following abbreviations are used in this manuscript:

ASL	American Sign Language
L1	First-language
L2	Second-language
